# Comparative Analysis of microRNA Binding Site Distribution and microRNA-Mediated Gene Expression Repression of Oncogenes and Tumor Suppressor Genes

**DOI:** 10.3390/genes13030481

**Published:** 2022-03-09

**Authors:** Shuangmei Tian, Jing Wang, Fangyuan Zhang, Degeng Wang

**Affiliations:** 1 Department of Environmental Toxicology, The Institute of Environmental and Human Health (TIEHH), Texas Tech University, 1207 Gilbert Dr., Lubbock, TX 79416, USA; shuangmei.tian@ttu.edu; 2 Department of Cancer Biology and Genetics, James Comprehensive Cancer Center, The Ohio State University Wexner Medical Center, Columbus, OH 43210, USA; jing.wang@osumc.edu; 3 Department of Mathematics and Statistics, Texas Tech University, Lubbock, TX 79409, USA; fangyuan.zhang@ttu.edu

**Keywords:** microRNA, miRNA binding sites, miRNA-regulated repression, tumor suppressor genes, oncogenes

## Abstract

MicroRNAs (miRNAs) are a family of short, noncoding RNAs that can regulate gene expression levels of over half of the human genome. Previous studies on the role of miRNAs in cancer showed overall widespread downregulation of miRNAs as a hallmark of human cancer, though individual miRNAs can be both tumor suppressive and oncogenic, and cancer genes are speculated to be more targeted by miRNA. However, the extents to which oncogenes and tumor suppressor genes (TSG) are controlled by miRNA have not been compared. To achieve this goal, we constructed lists of oncogenes and TSGs and compared them with each other, and with the whole protein-coding gene population, in terms of miRNA binding sites distribution and expression level changes upon genetic disruption of miRNA production. As expected, the results show that cancer gene mRNAs anchor more miRNA binding sites, and are under a higher degree of miRNA-mediated repression at both mRNA abundance and translation efficiency levels than the whole protein-coding gene population. Importantly, on average, TSG mRNAs are more highly targeted and regulated by miRNA than oncogene mRNAs. To the best of our knowledge, this is the first comparison of miRNA regulation of oncogenes and TSGs.

## 1. Introduction

MicroRNAs (miRNAs) are small (17–25 nucleotide (NT); on average 22 NT), endogenously-initiated, single-stranded noncoding RNAs that exist ubiquitously in animals, plants, and unicellular eukaryotes as key post-transcriptional regulators of gene expression [1,2,3,4]. In the canonical pathway of miRNA biogenesis in animals, miRNA genes are transcribed by RNA polymerase II (Pol II) as long primary transcripts (pri-miRNAs), processed to hairpin-structured precursor miRNAs (pre-miRNAs) by the nuclear Microprocessor complex (comprising Drosha and DGCR8), and then exported by exportin 5 from the nucleus into the cytoplasm [4]. In the cytoplasm, pre-miRNAs are cleaved by Dicer1 to double-stranded RNA duplexes, and subsequently loaded into Argonaute (AGO) proteins to form the RNA-induced silencing complex (RISC). The passenger strand of the miRNA duplex will be cleaved quickly, and the mature RISC is finally generated, comprising the one-stranded mature miRNA and AGO proteins. The miRNA in RISC guides target mRNA recognition through base-pairing with partially complementary sequences, mostly in the 3′-untranslated region (3′-UTR) of target mRNAs. The AGO proteins regulate translational repression and/or mRNA degradation by recruiting other effector proteins [5,6].

Partial complementarity between a miRNA and its target mRNAs’ UTR is sufficient for the miRNA to regulate the target mRNAs. Many mRNAs’ UTRs can have multiple conserved complementary sequence segments for different miRNA seeds; thus, one mRNA can have target sites for tens of miRNAs, and one miRNA can potentially regulate several hundreds of target mRNAs based on the computational prediction of miRNA target sites [7,8,9,10,11]. Over 60% of human protein-coding genes are predicted to have conserved miRNA target sites in their 3′-UTR [12]. These miRNA-target mRNA interactions form complex regulatory networks in cellular development, differentiation and homeostasis [13]. Therefore, it is not surprising that miRNAs regulation is involved in many key biological processes, such as animal development [14], immune response [15], neuronal function [16] and metabolic homoeostasis [17].

Dysregulation of miRNA biogenesis and/or function has been associated with the development and progression of numerous human diseases in the past decades. Specifically, studies on miRNA and cancers are growing explosively [18,19]. Many miRNAs were found to be expressed in human tumors differentially from normal tissues, and these tumor-associated miRNAs preferentially regulate protein-coding tumor suppressors and oncogenes [20,21]. This indicated that deregulation of miRNA interactions with cancer genes’ mRNAs might be one critical pathway leading to tumorigenesis. One speculated mechanism for miRNA association with cancer is that overexpression of oncogenic miRNAs, and/or deletion or silencing of tumor suppressive miRNAs promotes cancer pathogenesis by negatively regulating target tumor suppressor genes (TSG) and oncogenes, respectively [22,23], and this mechanism has been validated in many individual miRNA’s animal or in vitro experiments [24]. 

Nevertheless, the role of a specific miRNA in tumor initiation and development is often ambiguous due to the complexity of the miRNA–mRNA target relationship and the involvement of individual miRNA generally in multiple cellular pathways. Different families of miRNAs can show either oncogenic activity through repression of TSGs, or tumor suppressive activity by inhibiting the expression of oncogenes that control cell proliferation, differentiation or apoptosis. That is, one specific miRNA can be considered oncogenic in one scenario and tumor suppressive in another [25]. It is not feasible to experimentally study the whole set of miRNA interaction with target cancer gene mRNAs in any given single cancer type, as it is prohibitedly time-consuming and expensive. 

However, one overall observed trend is the widespread downregulation of miRNAs, generally considered a hallmark of human cancer [26]. Moreover, the development of next-generation sequencing (NGS) has accumulated massive amounts of sequencing data, which made it possible to analyze the interaction between cancer genes and miRNAs on a genome-wide scale. Thus, in this study, instead of focusing on individual miRNA–mRNA relationships, we performed an overall comparison of protein-coding genes, oncogenes, and TSGs. We utilized available miRNA target prediction resources and global gene expression data upon genetic disruption of miRNA production. We also assembled a group of well-constructed human cancer genes. Subsequently, we were able to investigate whether oncogenes or TSGs are more miRNA targeted—a major question complementing the overall downregulation of miRNA in cancer. Thus, the analysis fills a gap in the roles of miRNA in cancer.

## 2. Materials and Methods

### 2.1. Evolutionarily Conserved miRNA Binding Sites

The set of evolutionarily conserved human miRNA binding sites was developed by Agarwal et al. using context++ model prediction and downloaded from the TargetScan database 7.1 (June 2016 release) [10]. HGNChelper R package (version 0.8.1) and updated reference map via function getCurrentHumanMap were used to update obsolete gene symbols and historical aliases to current gene symbols maintained by The HUGO Gene Nomenclature Committee (HGNC) database [27]. The dataset contains 116,371 predicted miRNA binding sites in the 3′-UTRs of 12,436 human genes. Overlapping sites were counted as one site in the current analysis.

### 2.2. Comparative RNA-seq Analysis of Wildtype and Dicer1 Knockout (KO) Cells

As discussed in the Introduction, Dicer1 is a key RNase that generates mature miRNAs. Dicer1 KO cells should be miRNA production deficient and a good tool for studying miRNA-mediated mRNA regulation. Fortunately, Zheng et al. had studied the transcriptome differences between wild type and Dicer1 deficient mouse embryonic stem cells (mESCs) using the RNA-seq analysis [28]. Their data were deposited in the Gene Expression Omnibus (GEO) under accession number GSE55338. In their experiment, total polyadenylated RNA was isolated from WT (two biological replicates) and Dicer1-KO (three biological replicates) mESCs, and sequenced on the Hi-Seq 2000 Illumina platforms. DESeq29 was used to normalize reads between samples. We downloaded the dataset, selected those mRNAs that have at least two replicates with >1 normalized reads, and calculated the log2 fold change between WT and Dicer1-KO cells. Gene identifiers (ID) were converted to gene symbols and annotated with biotype by accessing the Ensembl database (release 104) via the R package BiomaRt (version 2.46.3) [29]. Only protein-coding genes were selected for expression analysis in the current study.

### 2.3. Comparative Polysome Profiling Analysis of Wildtype and Dicer1 KO HCT116 Cells

Polysome profiling analysis measures mRNA polysome association, i.e., translation activity. Fortunately, we have previously performed polysome profiling analysis of wild type and Dicer 1 KO HCT116 human cells. Detailed experimental information was described previously [30]. Briefly, cells were lysed. The nucleus was removed by microcentrifugation. The cytoplasmic extract was loaded on top of a 10–60% sucrose gradient, and then centrifuged in a Beckman SW41 rotor at 390,000× *g* at 4 °C for 2 h. The gradient was fractionated into 25 fractions. Light polysomes fractions (2- to 9-mer) and heavy polysomes fractions (10-mer or more) were collected, respectively, and associated RNA was extracted. One sample per condition was sequenced by RNA-seq on the BGISEQ-500 high-throughput sequencing platform. The sequencing data were deposited into GEO with the accession number GSE134818. The counts were converted to reads per kilobase of transcript, per million mapped reads (RPKM). To decrease the sequencing noise of low expression genes, only those genes whose RPKM > 0 for all samples and at least one sample’s RPKM > 1 were retained for the analysis in the current study. Protein-coding genes were identified and screened via the R package BiomaRt (version 2.46.3, Ensembl release 104), and their expression data were applied in the current study.

### 2.4. Compilation of Cancer Genes and miRNA Binding Sites, and Analysis of mRNA Polysome Association (Translation Activity) in miRNA-Production-Deficient Cells

To construct the cancer gene list, we adopted the approach by Sack et al. [31]. Briefly, as Sack et al. did, we integrated three previously published gene lists. First, Davoli et al. (2013) developed the Tumor Suppressor and Oncogenes (TUSON) Explorer bioinformatics method, and analyzed >8200 tumors of all types (such as Gliobloastoma, Low Grade Glioma, Breast Adenocarcinoma, Colorectal Adenocarcinoma, etc. For details of all analyzed tumor types, see their Table S1). They predicted both tissue specific cancer genes and pan-cancer genes (For detailed genes list, tumor types and TUSON Prediction scores, see their Table S4A,B) [32]. Second, Volgelstein et al. (2013) evaluated ~140 genes whose genetic alternations can “drive” tumorigenesis based on the genome-wide sequencing studies of 3284 tumors in representative human cancers (For detailed lists, see their Table S2A,B) [33]. Third, Futreal et al. (2004) compiled a list of cancer genes from published literature of genes that were mutated and causally implicated in cancer development (For detailed gene list and tumor types, see their Supplementary information S1) [34]. Our compiled oncogene list consists of the following: top 205 TUSON Explorer predicted pan-cancer oncogenes with q-value < 0.18 [32], 54 Mut-driver and 10 amplified driver oncogenes [33], and 227 dominantly acting genes [34]. Our compiled TSG list consists of the following: top 301 TUSON Explorer predicted pan-cancer TSGs with q-value < 0.18 [32], 71 Mut-driver and 3 homozygously deleted TSGs [33], and 64 recessively acting genes [34]. Gene symbols were checked by the HGNChelper R package. Upon removal of redundancy among the three sources, the list contains 409 oncogenes and 324 TSGs (Table S1A,B).

The data of predicted miRNA binding sites among 733 cancer genes were obtained from TargetScan database 7.1, and the distribution pattern was compared with total protein-coding genes. The mRNA expression data of the whole genome were obtained from GSE55338, and the expression changes of cancer genes between wildtype and Dicer knockout mESCs were compared with the expression changes of total non-cancer protein-coding genes.

For the comparative polysome profiling analysis [30], the HCT116 used in the analysis is a widely used human colorectal carcinoma cell line. Thus, we incorporated the colorectal-specific oncogenes and TSGs predicted by TUSON into this analysis [32]. This cancer gene list contains 413 oncogenes and 415 TSGs (Table S1C,D). Polysome association was compared in a pairwise manner among the cancer genes, the oncogenes, the TSGs and the total non-cancer protein-coding genes. The comparison of gene groups, as opposed to individual genes under different conditions, offset the lack of biological replicates in the polysome profiling analysis.

### 2.5. Computer Software

The open source software package R (version 4.0.2) was used for data analysis and plotting. The Mann-Whitney-Wilcoxon tests were performed with the wilcox.test method since the expression data were non-normal.

### 2.6. Overall Study Design

**Figure 1:**
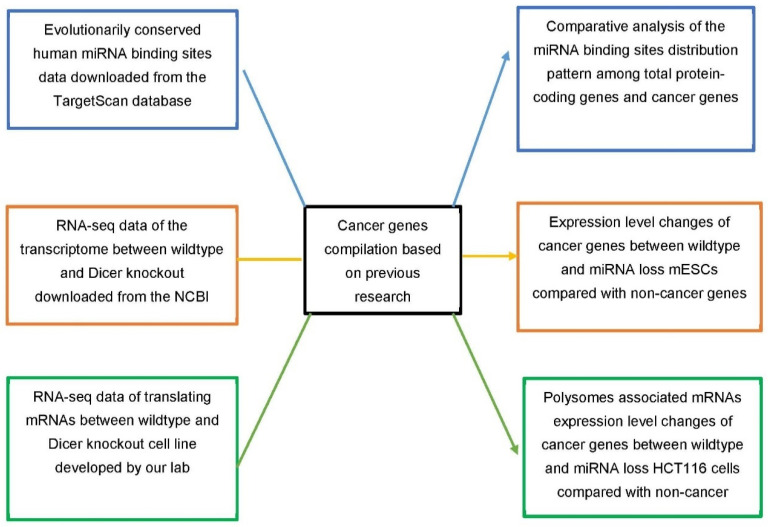
Schematic representation of the experimental design for the comparison of miRNA-mediated gene expression repression of oncogenes and TSGs.

The overall design or workflow of this analysis is illustrated in Figure 1. The blue textboxes and arrows denote comparative analysis of miRNA binding site distribution in the whole protein-coding gene population, the cancer genes, the oncogenes and TSGs. The orange textboxes and arrows denote the analysis of wild-type versus Dicer1 knockout mice RNA-seq dataset, and the green textboxes and arrows denote the analysis of wild-type versus Dicer1knockout polysome profiling dataset. Cancer genes are excluded from the general protein-coding gene population in our analysis.

## 3. Results

### 3.1. Distribution of miRNA Binding Sites among Cancer Genes

Our previous study has shown that conserved miRNA binding sites distribute unevenly among human transcriptome, following the so-called scale-free distribution that is applicable to many molecular and cellular phenomena [35,36,37]. Only a small number of mRNAs contain extraordinarily large numbers of miRNA binding sites, and a small number of miRNAs can target a large number of mRNAs [38]. The uneven distribution pattern of predicted miRNA target sites is also found in the mouse genome [39], and orthologous human and mouse mRNA have highly similar miRNA target sites counts [30]. 

The TSGs and oncogenes investigated in the current study function in many known cellular pathways and are enriched for hubs within the human gene network [32]. Hub genes are much more highly connected than average, and their mRNAs tend to possess longer 3′ UTR and higher density of miRNA target sites [40]. Therefore, we speculated that cancer genes have more enriched miRNA binding sites than average protein-coding genes, and the results confirmed our speculation. 

**Figure 2:**
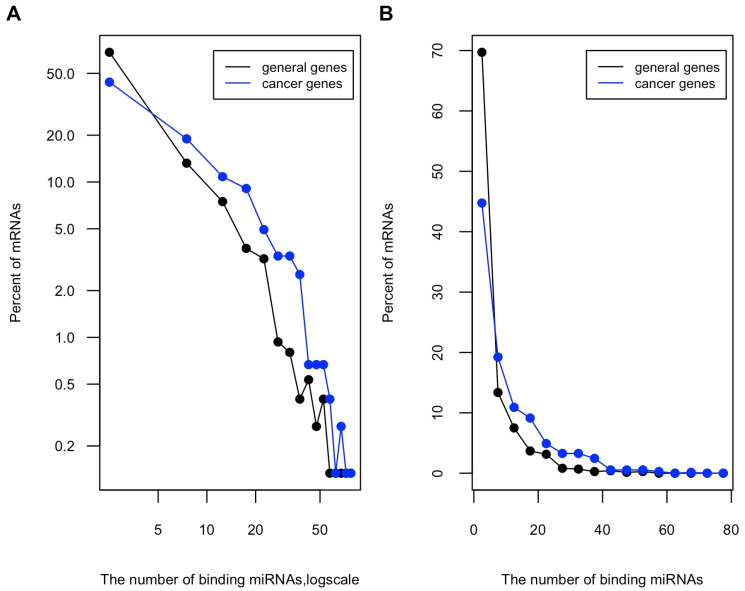
Distribution of miRNA binding sites among general protein-coding genes and the cancer genes. The number of miRNA target sites among 733 cancer genes (409 oncogenes and 324 TSGs, see Materials and Methods), and 733 general protein-coding genes randomly selected from the human genome were obtained from TargetScan 7.1 database as described before. The histograms of miRNA binding sites number are shown in (**A**) log-log plot and (**B**) linear plot.

**Figure 3:**
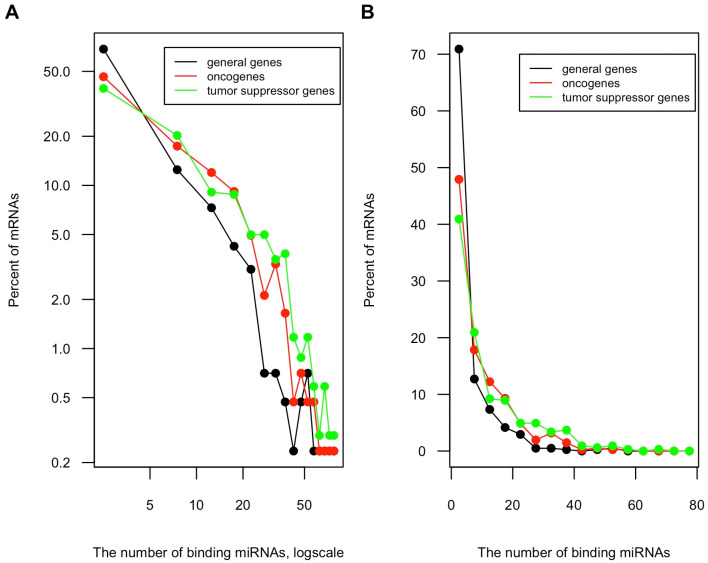
Distribution of miRNA binding sites among general protein-coding genes, the oncogenes and the TSGs. The number of miRNA target sites among 409 oncogenes, 324 TSGs and 409 protein-coding genes randomly selected from the human genome were obtained from TargetScan 7.1 database as described before. The histograms of miRNA binding sites number are shown in (**A**) log-log plot and (**B**) linear plot.

About 60% of all the whole protein-coding gene population have at least one miRNA target site. This percentage increases to over 80% (598 of 733) among cancer genes. The distribution pattern of predicted miRNA target sites among these cancer genes is similarly uneven as that of the whole protein-coding gene population. However, cancer genes have higher portions of highly targeted genes than general protein-coding genes (Figure 2A,B). Furthermore, we compared the distribution of predicted miRNA target sites in oncogenes and TSGs. Figure 3A,B show that TSGs are more enriched for miRNA sites, having higher portions of genes that harbor more than 20 miRNA targeted sites than oncogenes. 

**Figure 4:**
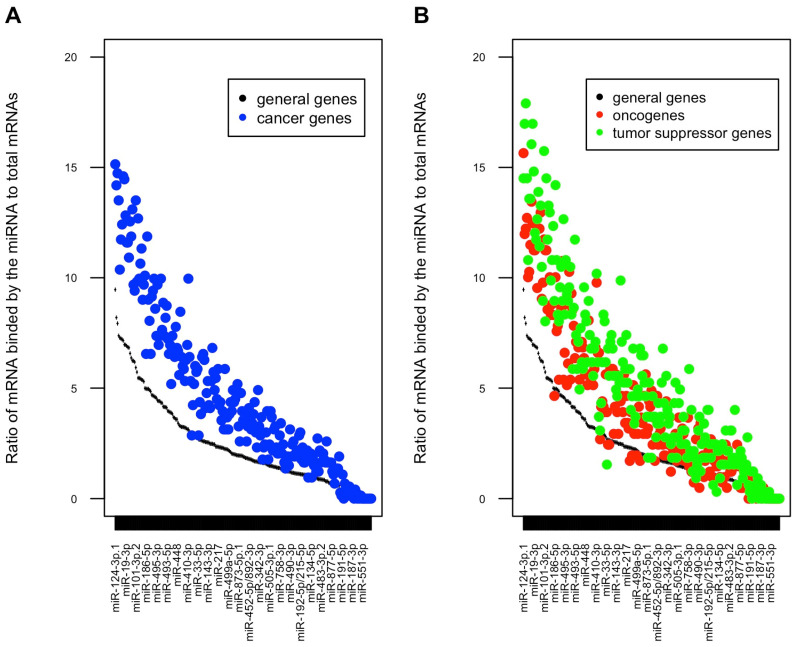
Ratio/Proportion of cancer genes (**A**), and TSGs/oncogenes (**B**) targeted by individual miRNAs as predicted in TargetScan 7.1 database.

There are 225 miRNA families in the TargetScan 7.1 conserved target sites prediction, and 212 of them have target genes in the list of 733 cancer genes. As shown in Figure 4A, different miRNA families have different numbers of target genes, and those miRNAs that have high ratios of target genes among the whole human protein-coding gene population also have high ratios of target genes among 733 cancer genes. Almost all miRNAs have higher ratios among the cancer genes than the general protein-coding genes (Figure 4A), and higher ratios among the TSGs than the oncogenes (Figure 4B).

Overall, these results demonstrate that cancer genes have more miRNA target sites than general protein-coding genes, and TSGs harbor more miRNA target sites than oncogenes. Consistently, we observed higher percentages of cancer genes than general protein-coding genes targeted by individual miRNAs, and higher ratios of targeted TSGs than oncogenes. 

### 3.2. The TSG mRNA Abundances Are Depressed More than Oncogene mRNA Abundances in DICER1 KO mESCs

Generally, genes harboring higher numbers of target sites for the same or different miRNAs are under stronger regulated repression of miRNAs [41,42]. Our previous study supported this notion by showing that the global expression changes due to the loss of miRNA expression were correlated with miRNA binding sites counts [30,38]. As discussed above, cancer genes tend to have more miRNA target sites than general protein-coding genes, especially the TSGs (Figure 2, Figure 3 and Figure 4). Therefore, we hypothesized that miRNAs have a higher repression capacity on cancer genes than general protein-coding genes, and the mRNA expression level of cancer genes would be relieved to higher degrees upon miRNA loss.

**Figure 5:**
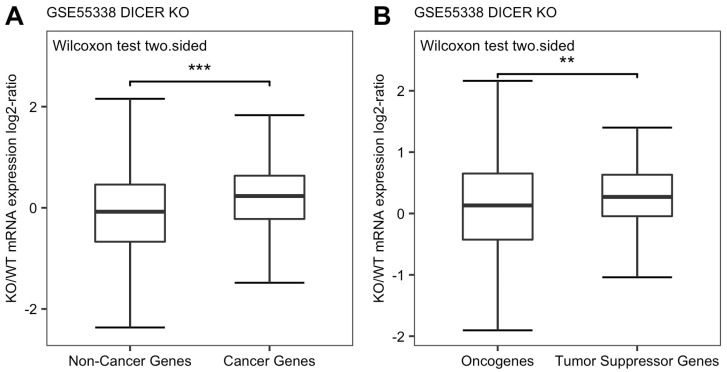
(**A**) Average expression changes of total protein-coding genes and 733 cancer genes in Dicer knockout mESCs (*p*-value < 2.2 × 10^−16^ by two-sided Wilcoxon rank-sum test). (**B**) Average expression changes of 409 oncogenes and 324 TSGs in Dicer knockout mESCs (*p*-value = 0.0094 by two-sided Wilcoxon rank-sum test). (“**” indicates *p* < 0.01, “***” indicates *p* < 0.001).

To confirm this hypothesis, we searched available RNA-seq data that compared the difference of transcriptome between wildtype and Dicer1 KO cells on NCBI GEO database, and found that the study of Zheng et al. provided such information as discussed on Materials and Methods [28]. We explored their data (GEO accession number GSE55338) and compared expression changes of cancer genes with total protein-coding genes. It showed that the log2 fold change of 733 cancer genes between Dicer1 KO and WT was higher than zero, and significantly higher than total protein-coding genes (*p*-value < 2.2 × 10^−16^, Figure 5A). It means that the mRNA expression levels of cancer genes are increased in Dicer1 KO mESCs, and the elevation degree is significantly higher than total protein-coding genes, whose log2 fold change of KO/WT was about zero. We next compared the expression changes between oncogenes and TSGs and found that the derepression of TSGs was significantly higher than that of oncogenes (*p*-value = 0.0094, Figure 5B), although their expressions were both increased in Dicer KO mESCs. These results suggest that the mRNA levels of cancer genes are under stronger control by miRNA than the general protein-coding genes, and among all cancer genes, miRNA down-regulates TSGs more than oncogenes.

In addition, we observed that cancer genes exhibit a more condensed distribution than the whole protein-coding gene population (Figure 5A), and that TSGs exhibit a more condensed distribution than the oncogenes (Figure 5B). The results suggest that the whole protein-coding gene population, the oncogenes and the TSGs are under sequentially tighter mRNA expression control. There must be other non-miRNA regulatory mechanisms for oncogene and TSG expression. 

### 3.3. Translating TSG mRNA Abundances Are Depressed More than Those of Oncogene mRNAs in Dicer Knockout Human HCT116 Cells

As discussed in the Introduction, miRNA can regulate gene expression at the translational level as well. Polysome profiling has been the most common technique for studying translating mRNAs specifically [43]. During polysome profiling, actively translating mRNAs associated with polysomes (multiple ribosomes) are separated from untranslating mRNAs (“free mRNAs” or monosomes) through sucrose density gradient centrifugation. This fractionation can also separate more efficiently translated mRNAs associated with heavy polysomes from those associated with light polysomes [44]. After isolation of RNA from the fractions, the distribution of specific mRNAs can be analyzed with RNA-seq [45].

**Figure 6:**
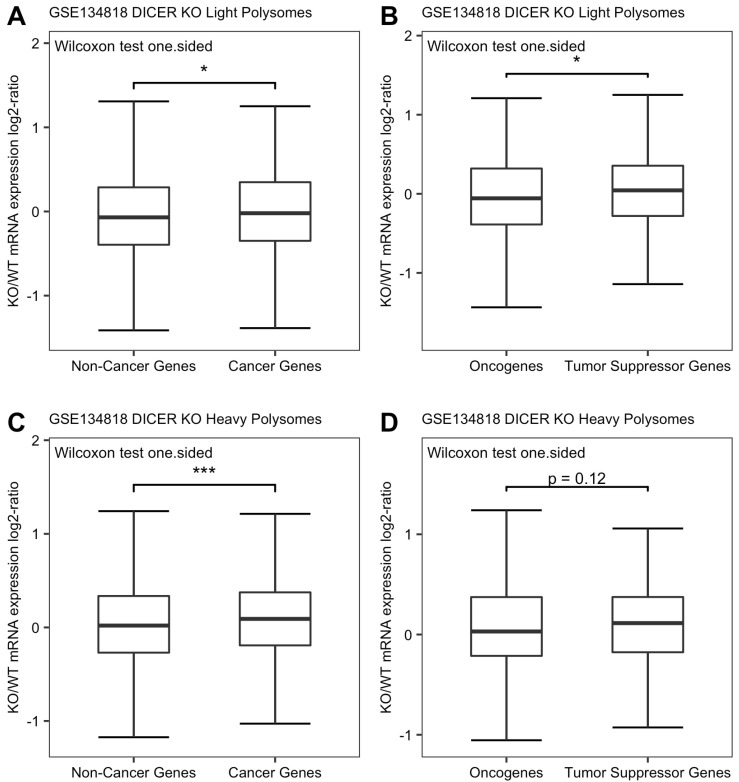
(**A**) Average light polysomes associated mRNA expression changes of total protein-coding genes and 828 cancer genes in Dicer1 knockout human HCT116 cells (*p*-value = 0.015 by one-sided Wilcoxon rank-sum test). (**B**) Average light polysomes associated mRNA expression changes of 413 oncogenes and 415 TSGs in Dicer1 knockout human HCT116 cells (*p*-value = 0.032 by one-sided Wilcoxon rank-sum test). (**C**) Average heavy polysomes associated mRNA expression changes of total protein-coding genes and 828 cancer genes in Dicer1 knockout human HCT116 cells (*p*-value = 0.00086 by one-sided Wilcoxon rank-sum test). (**D**) Average heavy polysomes associated mRNA expression changes of 413 oncogenes and 415 TSGs in Dicer1 knockout human HCT116 cells (*p*-value = 0.12 by one-sided Wilcoxon rank-sum test). (“*” indicates *p* < 0.05, “***” indicates *p* < 0.001).

Fortunately, relevant polysome profiling was previously conducted by us as described in Materials and Methods [30]. We analyzed the data to assess the effects of miRNA loss on actively translating mRNAs abundance. The results showed a similar pattern among cancer genes, oncogenes, TSGs and the whole protein-coding gene population in Dicer1 KO human HCT116 cells, even though the statistical power of our analysis was likely reduced by the lack of biological replications in the NGS analysis. The log2 fold change of KO/WT of either light polysomes associated mRNA or heavy polysomes associated mRNA was significantly higher for cancer genes than for corresponding total protein-coding genes, respectively, (*p*-value = 0.015 by one-side Wilcoxon test, Figure 6A; *p*-value = 0.00086 by one-sided Wilcoxon test, Figure 6C). Among light polysomes associated mRNAs, TSGs had a significantly higher degree of increase than oncogenes, and the distribution of their expression changes was more condensed than that of oncogenes (Figure 6B). The same pattern was observed for heavy polysome associated mRNA abundance (Figure 6D). Therefore, these results support the notions that actively translating mRNAs of cancer genes are upregulated more than the general protein-coding genes upon miRNA loss, and that TSGs are derepressed at a higher capacity than oncogenes. 

## 4. Discussion

We compared the levels of miRNA control of oncogenes and TSGs in a global manner. This analysis complements the observed overall trend of miRNA downregulation in cancer. Our results showed that, in general, the TSGs were under higher degree of regulation by miRNA than the oncogenes (Figure 3B, Figure 4B and Figure 5B and Figure 6B,D). This new insight into the relationship between miRNA and cancer was enabled by the global manner of this analysis. Otherwise, this global pattern would be submerged, as described below, by the noises caused by the complexity of individual miRNA-cancer and miRNA-target relationship. 

High level of complexity is intrinsic to the miRNA-cancer and miRNA-target relationships. Each miRNA typically targets many genes, and individual genes can be targeted by multiple miRNAs. MiRNA-targeting activities can induce intensive feedback effects, either directly or through the interaction with transcription factors [46]. This is especially applicable to cancer genes due to the enrichment of miRNA binding sites among their mRNAs. An individual miRNA might target both oncogene and TSG mRNAs. The observed global pattern would be lost if one focused on individual miRNAs. For example, in the current study, miR-21 targeted 2% total protein-coding genes, whereas 6.6% TSGs and 3% oncogenes, respectively, though miR-21 indeed has the ability of targeting key tumor-suppressive pathways in tumor tissues [47]. We also observed that several well-known tumor suppressive miRNAs, such as miR-34 [48], miR-200 [49] and miR-326 [50], targeted more oncogenes than TSGs although total miRNAs targeted more TSGs than oncogenes on average. In other words, this is a typical case of the whole forest versus individual trees argument. There is a clear need for global analysis in analyzing miRNA-cancer and miRNA-target relationships.

Our results are consistent with the notion that the number and arrangement of miRNA binding sites correlates with the capacity of miRNA-mediated gene repression, which is derived from multiple experimental and computational analysis [30,41,51]. Cancer gene mRNAs are more enriched for miRNA binding sites [38,41]. TSG mRNAs contain more sites than oncogene mRNAs. Consistently, cancer genes exhibited higher levels of mRNA expression derepression than the general protein-coding gene population, and TSGs exhibited higher levels than oncogenes. 

However, questions remain regarding the functional advantages of miRNA regulatory actions, i.e., why the cells maintain such a regulatory mechanism. For instance, whether miRNAs regulate gene repression primarily through promoting mRNA degradation or impacting translation activity remains controversial. Some studies argue that mRNA degradation can explain a larger fraction of miRNA-regulated repression than changes in translational efficiency [52,53]. However, there are other studies demonstrating that miRNAs induced translational repression precedes mRNA destabilization [54,55,56] and that the impact of miRNA on translation alone can recapitulate a large portion of the downstream molecular and phenotypic effects associated with miRNA loss [57]. Additionally, it is unclear why the cells produce mRNAs that are quickly degraded and not used for protein production, which seems very wasteful of the building blocks and other metabolic resources. Finally, but not the least, why is the repression at both mRNA abundance and translation activity levels so moderate? A complete repression seems more logical.

Our results add to the confusion. The overall miRNA downregulation in cancer seems contradictory to the higher levels of miRNA regulation of the TSGs than the oncogenes. If the functional consequence of miRNA regulatory actions is truly suppression of the targeted genes, miRNA expression should exhibit the opposite pattern to the target. MiRNA should be up-regulated, instead of being down-regulated as the TSGs, in cancer. We believe the answer to this contradiction awaits a better understanding of the cellular advantages of miRNA regulatory actions.

To conclude, our study confirms the notion that cancer genes are more tightly regulated by miRNA than the general protein-coding gene population. More importantly, we report, for the first time, that TSGs are miRNA-controlled to a higher degree than oncogenes in terms of both miRNA binding site distribution and expression level changes upon genetic disruption of miRNA production. Further investigations are needed to reconcile this observation and the overall miRNA down-regulation in cancer and advance our understanding of the regulatory roles of miRNA.

## Data Availability

Our expression data were deposited in the GEO database (accession number: GSE134818).

## Supplementary Materials

The following are available online at https://www.mdpi.com/article/10.3390/genes13030481/s1. Table S1 as an Excel file with 4 spreadsheets: (A) List of Oncogenes Used for MiRNA Binding Sites and GSE55338 mESCs Gene Expression Analysis; (B) List of Oncogenes Used for MiRNA Binding Sites and GSE55338 mESCs Gene Expression Analysis; (C) List of Oncogenes Used for GSE134868 HCT116 Cells Gene Expression Analysis; and (D) List of TSGs Used for GSE134868 HCT116 Cells Gene Expression Analysis. Table S2 as an Excel file with 4 spreadsheets, each containing top 100 ranked genes according to the specified ranking criteria.
