# Supplementary figures and images for: Comparative Analysis of microRNA Binding Site Distribution and microRNA-Mediated Gene Expression Repression of Oncogenes and Tumor Suppressor Genes
Source: Genes (Basel). 2022 Mar 9;13(3):481. doi: 10.3390/genes13030481 (PMC8953695; doi:10.3390/genes13030481)

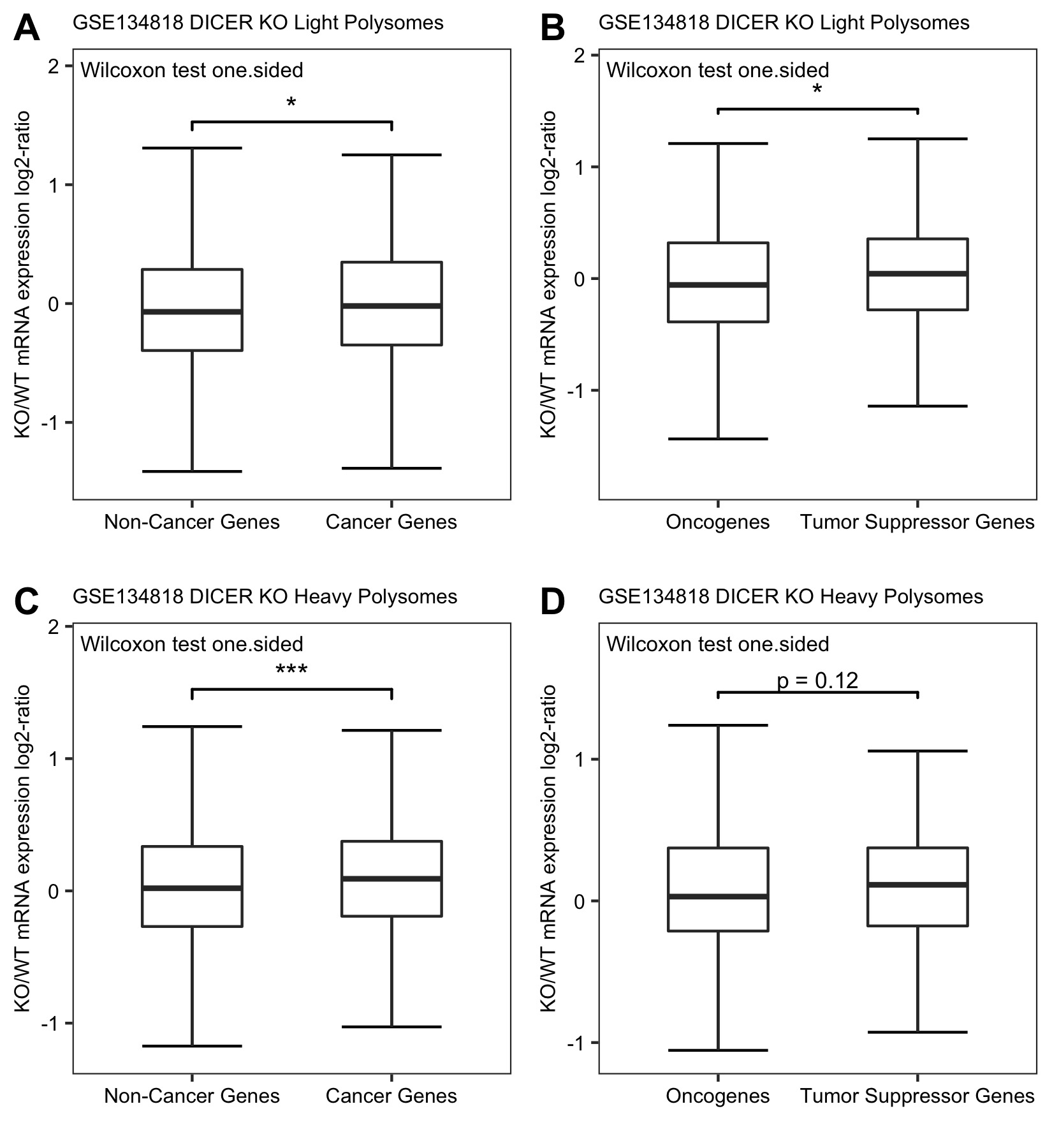

Supplement: Supplementary file 1 [file genes-13-00481-s001.zip › Revised Figs&Tables/Figure5.jpeg]

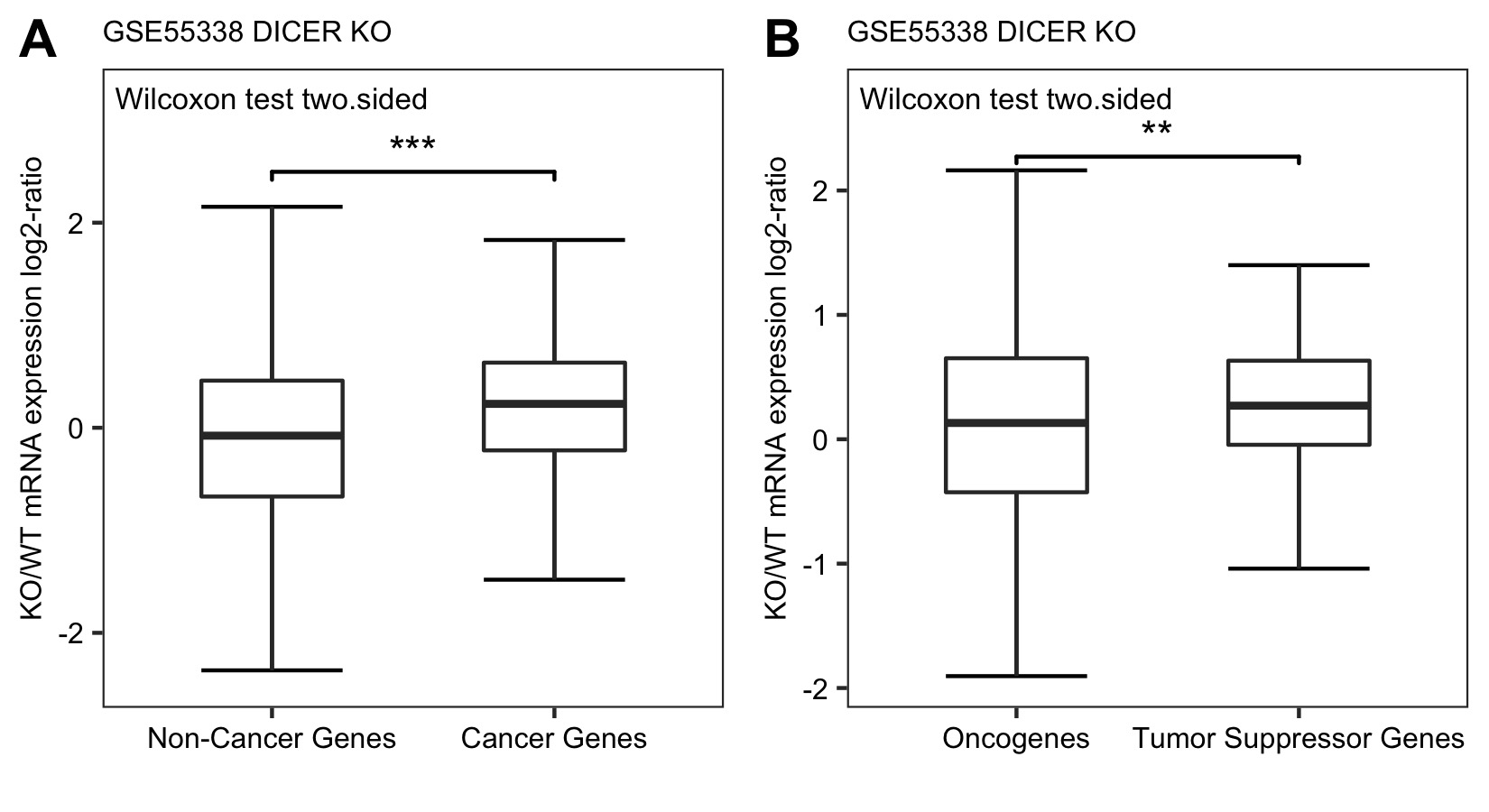

Supplement: Supplementary file 1 [file genes-13-00481-s001.zip › Revised Figs&Tables/Figure4.jpeg]

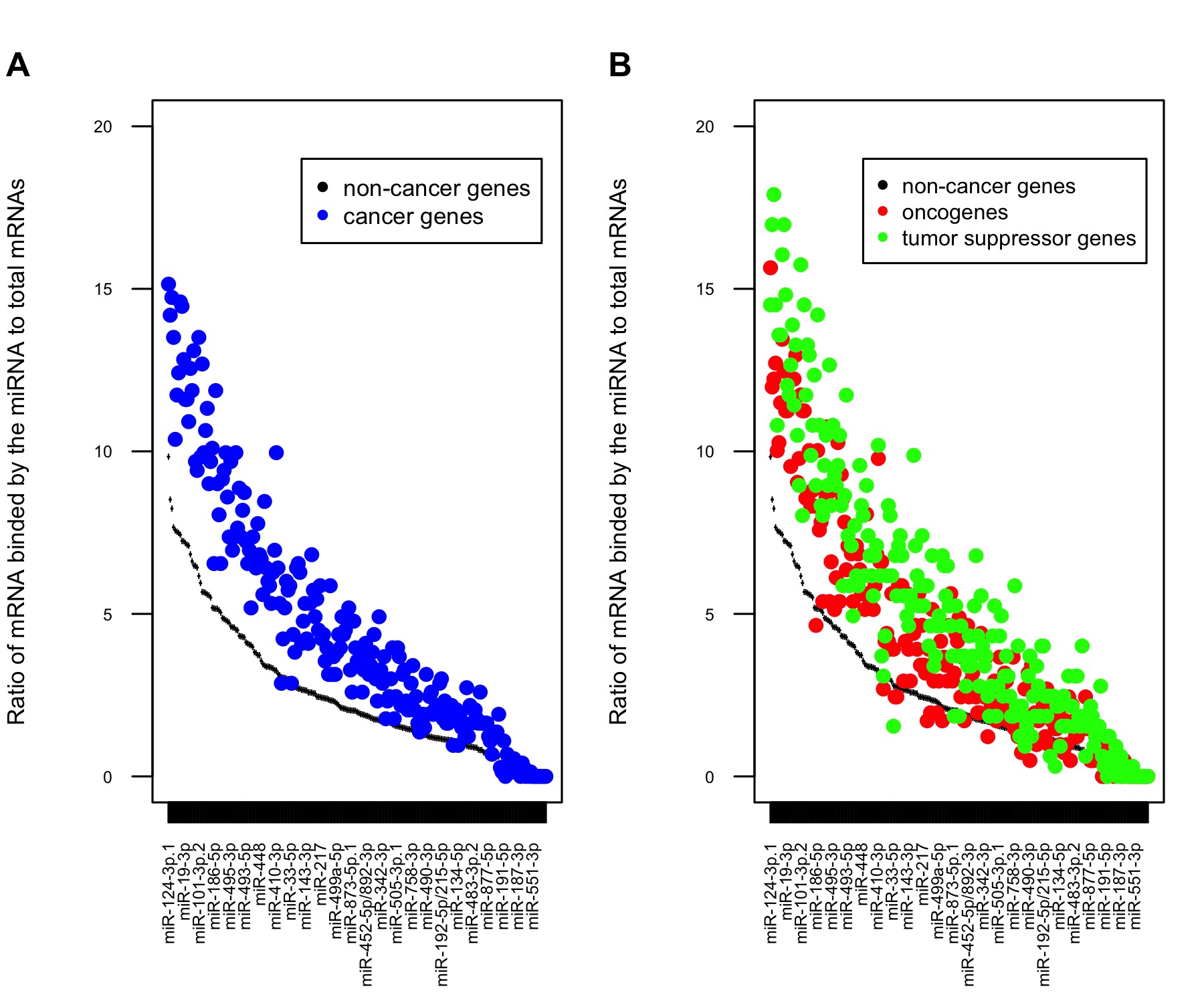

Supplement: Supplementary file 1 [file genes-13-00481-s001.zip › Revised Figs&Tables/Figure3.jpg]

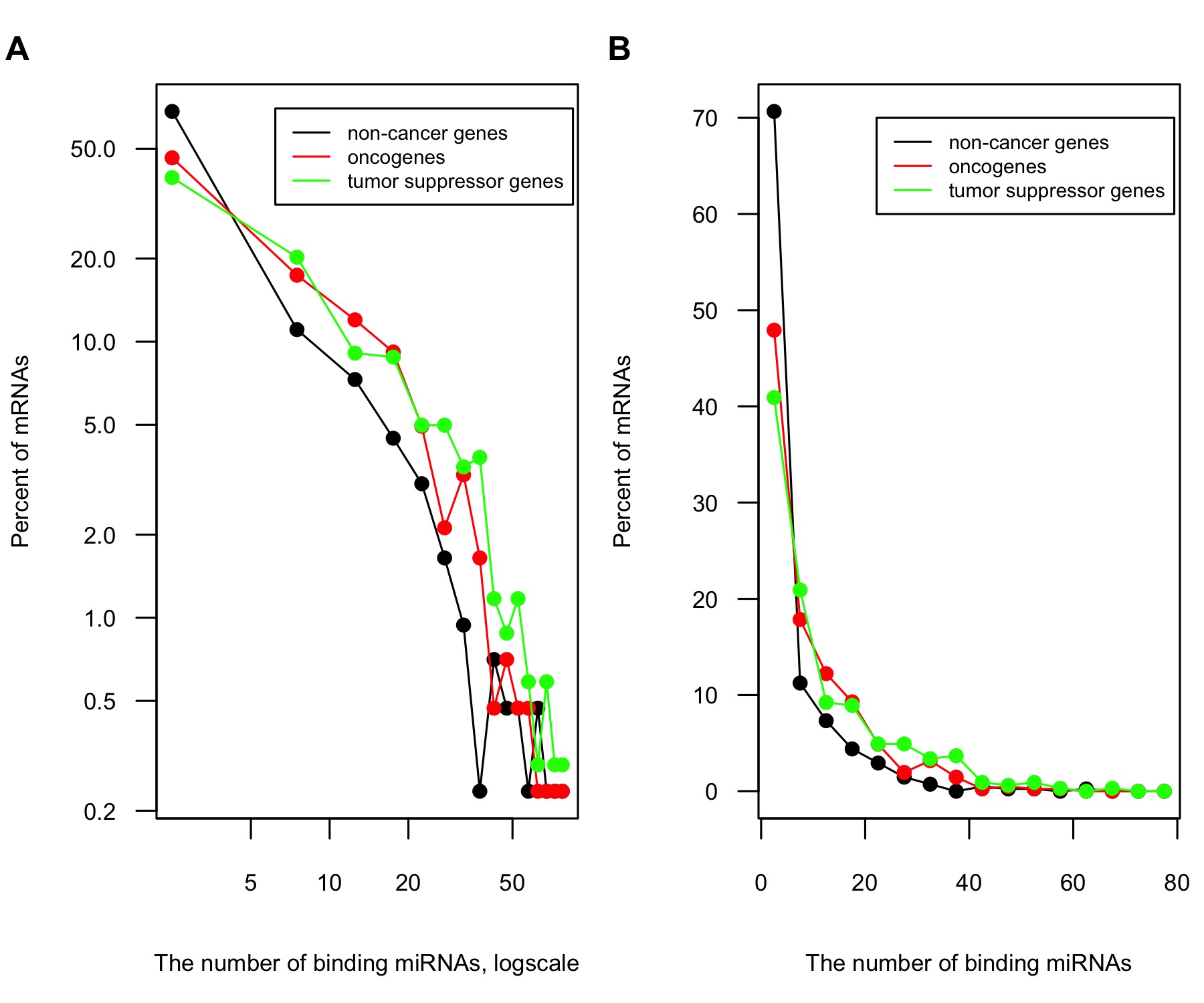

Supplement: Supplementary file 1 [file genes-13-00481-s001.zip › Revised Figs&Tables/Figure2.jpg]

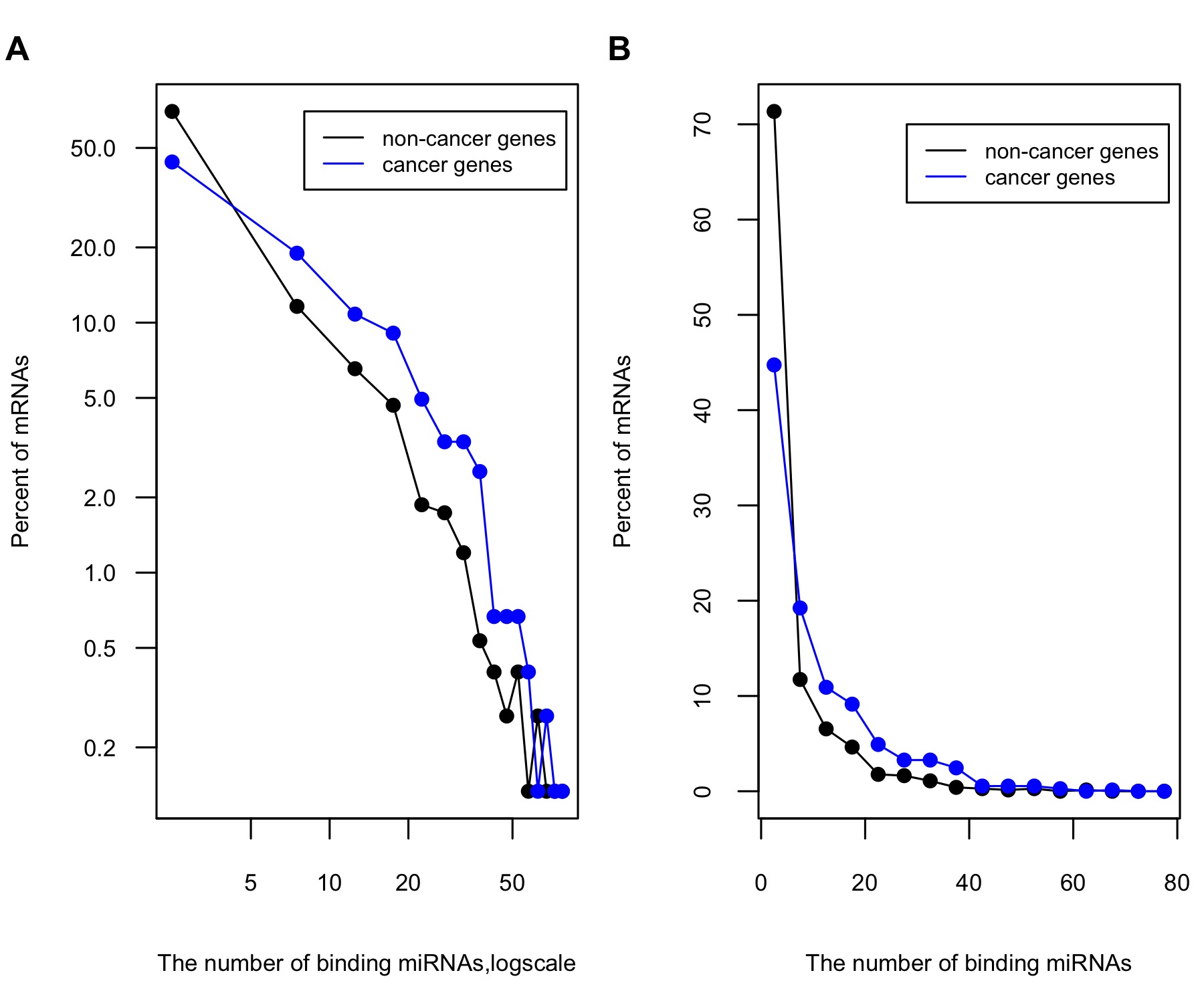

Supplement: Supplementary file 1 [file genes-13-00481-s001.zip › Revised Figs&Tables/Figure1.jpg]
